# Suitability of the Unified Theory of Acceptance and Use of Technology 2 Model for Predicting mHealth Acceptance Using Diabetes as an Example: Qualitative Methods Triangulation Study

**DOI:** 10.2196/34918

**Published:** 2022-03-09

**Authors:** Patrik Schretzlmaier, Achim Hecker, Elske Ammenwerth

**Affiliations:** 1 Institute of Medical Informatics UMIT – Private University for Health Sciences, Medical Informatics and Technology Hall in Tirol Austria; 2 Institute for Management and Economics in Healthcare UMIT – Private University for Health Sciences, Medical Informatics and Technology Hall in Tirol Austria; 3 DBU Digital Business University of Applied Sciences Berlin Germany

**Keywords:** mHealth, mobile health, mobile apps, diabetes mellitus, technology acceptance, UTAUT2, mobile phone

## Abstract

**Background:**

In recent years, the use of mobile health (mHealth) apps to manage chronic diseases has increased significantly. Although mHealth apps have many benefits, their acceptance is still low in certain areas and groups. Most mHealth acceptance studies are based on technology acceptance models. In particular, the Unified Theory of Acceptance and Use of Technology 2 (UTAUT2) model was developed to predict technology acceptance in a consumer context. However, to date, only a few studies have used the UTAUT2 model to predict mHealth acceptance and confirm its suitability for the health sector. Thus, it is unclear whether the UTAUT2 model is suitable for predicting mHealth acceptance and whether essential variables for a health-related context are missing.

**Objective:**

This study aims to validate the suitability of UTAUT2 for predicting mHealth acceptance.

**Methods:**

In this study, diabetes was used as an example as mHealth apps are a significant element of diabetes self-management. In addition, diabetes is one of the most common chronic diseases affecting young and older people worldwide. An explorative literature review and guided interviews with 11 mHealth or technology acceptance experts and 8 mHealth users in Austria and Germany were triangulated to identify all relevant constructs for predicting mHealth acceptance. The interview participants were recruited by purposive sampling until theoretical saturation was reached. Data were analyzed using structured content analysis based on inductive and deductive approaches.

**Results:**

This study was able to confirm the relevance of all exogenous UTAUT2 constructs. However, it revealed two additional constructs that may also need to be considered to better predict mHealth acceptance: *trust* and *perceived disease threat*.

**Conclusions:**

This study showed that the UTAUT2 model is suitable for predicting mHealth acceptance. However, the model should be extended to include 2 additional constructs for use in the mHealth context.

## Introduction

### Background

Mobile health (mHealth) apps are essential for effective self-management of chronic diseases such as diabetes [1,2]. In this context, mHealth describes mHealth technologies such as diabetes apps [3] and continuous glucose monitoring (CGM) systems [4] to support diabetes self-management and patient health [5-11]. The use of mHealth apps for diabetes self-management leads to more frequent monitoring of blood glucose levels and lower long-term glucose levels [12]. However, many patients do not use mHealth apps as they do not see the necessity or are satisfied with their current management [13]. Despite the potential and relevance of mHealth apps in chronic disease management, they are still used insufficiently [14].

An important aspect that determines the use of mHealth apps is their acceptance [15,16]: “User acceptance can be defined as the demonstrable willingness within a user group to employ information technology for the tasks it is designed to support" [17]. However, the acceptance of mHealth apps is still low in certain areas and groups [5,18-23]. For example, for type 2 diabetes, the acceptance of mHealth apps is low [12,24].

User acceptance often determines the success or failure of technical apps [25]. For predicting the acceptance of mHealth users, technology acceptance models are used [16,25]. These models are essential as they combine various theories from psychology and sociology to explain and predict technology acceptance and use [26].

We used diabetes as an example to investigate this issue as mHealth apps are a significant element of diabetes self-management [2,4,7].

In addition, diabetes is one of the most common chronic diseases, affecting approximately 463 million people worldwide between the ages of 20 and 79 years in 2019 [27]. Most patients (approximately 90%) have type 2 diabetes [27], where effective self-management can have a significant impact on improving patient health [5,6].

Many mHealth apps such as smartphone apps, blood glucose sensors (CGM), and others are used by patients with type 1 and type 2 diabetes in their self-management.

Therefore, we wanted to investigate the following research question in the field of mHealth self-management in diabetes: is the Unified Theory of Acceptance and Use of Technology 2 (UTAUT2) model suitable for predicting mHealth acceptance using diabetes as an example?

If the UTAUT2 model was better adapted to the needs of mHealth acceptance, the reasons for use or rejection of mHealth apps could thus be better predicted and more easily taken into account in new developments. This would help increase the use of mHealth self-management apps among people who are chronically ill, thereby improving their health.

### Theoretical Background

In health informatics, the Technology Acceptance Model (TAM), UTAUT, and UTAUT2 have proven to be suitable models for acceptance research [28-30]. These models consider constructs that influence the acceptance of technology to predict its use [28].

The TAM was developed in the late 1980s and provided the basis for further technology acceptance models [16,25]. It focuses on understanding why users accept or reject information technology (IT) systems and how their design influences acceptance [25]. The TAM hypothesizes that *perceived usefulness* and *perceived ease of use* are essential for the *attitude toward using*, which is a dominant factor of *behavioral intention to use* and can be interpreted as technology acceptance [25,28,31].

In 2003, the UTAUT model was published to present a unified model that synthesizes the diversity of acceptance models [16]. The basis of the UTAUT model is the analysis and comparison of 8 technology acceptance models (eg, Theory of Planned Behavior, TAM, and Innovation Diffusion Theory [16]). The UTAUT model aims to evaluate the likelihood of success of new technologies and understand the critical acceptance factors to proactively define measures to ensure that systems are accepted and used [16]. It uses the four central constructs of performance expectancy, effort expectancy, social influence, and facilitating conditions, moderated by gender, age, experience, and voluntariness of use, as direct determinants of behavioral intention and use behavior [16].

To date, mHealth acceptance studies have mainly used the TAM [32-35] and UTAUT [19,36,37] model or combinations of both [38,39]. Although the TAM was developed to predict the acceptance of IT systems [25], the UTAUT model focused on behavioral intention and technology use in organizational contexts [16].

In contrast, the focus of the UTAUT2 model, which was developed as an extension of the UTAUT model, is to predict technology acceptance in consumer use contexts [26]. Therefore, additional constructs such as hedonic motivation, price value, and habit were added [26].

**Figure 1:**
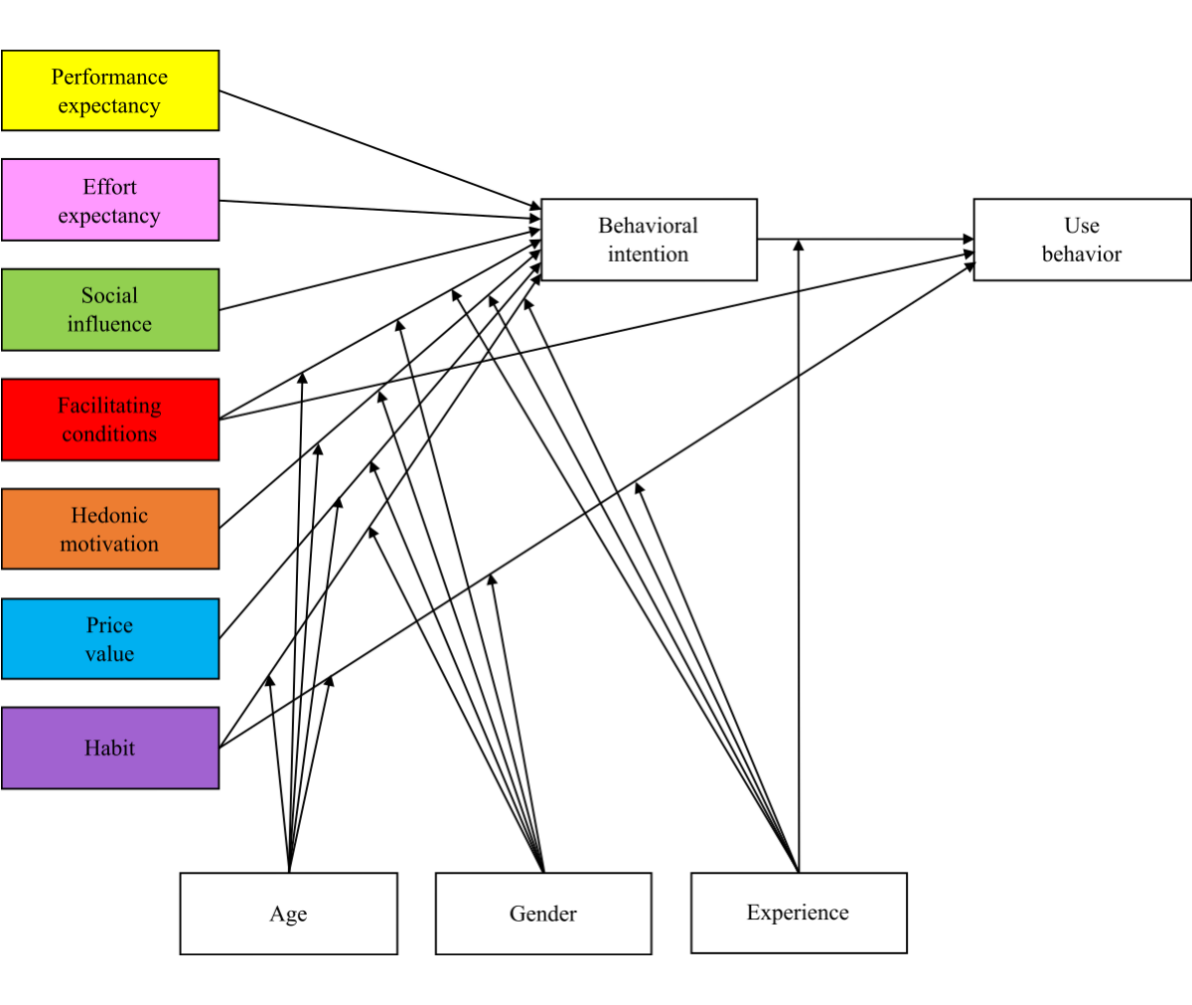
The Unified Theory of Acceptance and Use of Technology 2 model, adapted from a study by Venkatesh et al [26].

Figure 1 shows the UTAUT2 model developed by Venkatesh et al [26], with its exogenous constructs (colored boxes) of performance expectancy, effort expectancy, social influence, facilitating conditions, hedonic motivation, price value, and habit. It also shows the relationships between these exogenous constructs and the endogenous constructs of behavioral intention and use behavior [26]. Some of these relationships are moderated by age, gender, and experience [26].

As this study focuses on the suitability of the UTAUT2 model for predicting mHealth acceptance, Textbox 1 shows the definitions of the exogenous UTAUT2 constructs only, adapted from the study by Venkatesh et al [26].

**Exogenous Unified Theory of Acceptance and Use of Technology 2 constructs adapted from a study by Venkatesh et al [26].**

**Performance expectancy**

- “Degree to which using a technology will provide benefits to consumers in performing certain activities” [26]

**Effort expectancy**

- “Degree of ease associated with consumers’ use of technology” [26]

**Social influence**

- “Extent to which consumers perceive that important others (e.g. family and friends) believe they should use a particular technology” [26]

**Facilitating conditions**

- “Refer to consumers’ perceptions of the resources and support available to perform a behavior” [26]

**Hedonic motivation**

- “The fun or pleasure derived from using a technology” [26]

**Price value**

- “Consumers’ cognitive tradeoff between the perceived benefits of the applications and the monetary cost for using them” [26]

**Habit**

- “The extent to which people tend to perform behaviors automatically because of learning” [26]

Therefore, the UTAUT2 model seems appropriate specifically for mHealth technologies as it focuses on individuals and their needs [26,40]. This is visible, for example, in the construct of hedonic motivation, which has been described in some scientific articles as particularly important for consumers of a product or technology [26,41,42].

However, to date, only a few mHealth acceptance studies have used the UTAUT2 model, and out of the studies using it, some showed that primarily health-related factors such as health conditions, health consciousness, and health concerns are missing from the technology acceptance model [41,43,44]. These are particularly relevant for patients with chronic diseases who are using mHealth apps. In this context, mHealth acceptance may depend not only on fun or habit but also on the perceived threat of disease and perceived data security [18,19]. However, these aspects are not covered in the UTAUT2 model.

## Methods

We followed the 32-item COREQ (Consolidated Criteria for Reporting Qualitative Research) checklist [45].

### Design

We used a qualitative research design and triangulated an explorative literature review with guided interviews. The objective was to identify the main categories of mHealth acceptance in the field of diabetes self-management.

**Figure 2:**
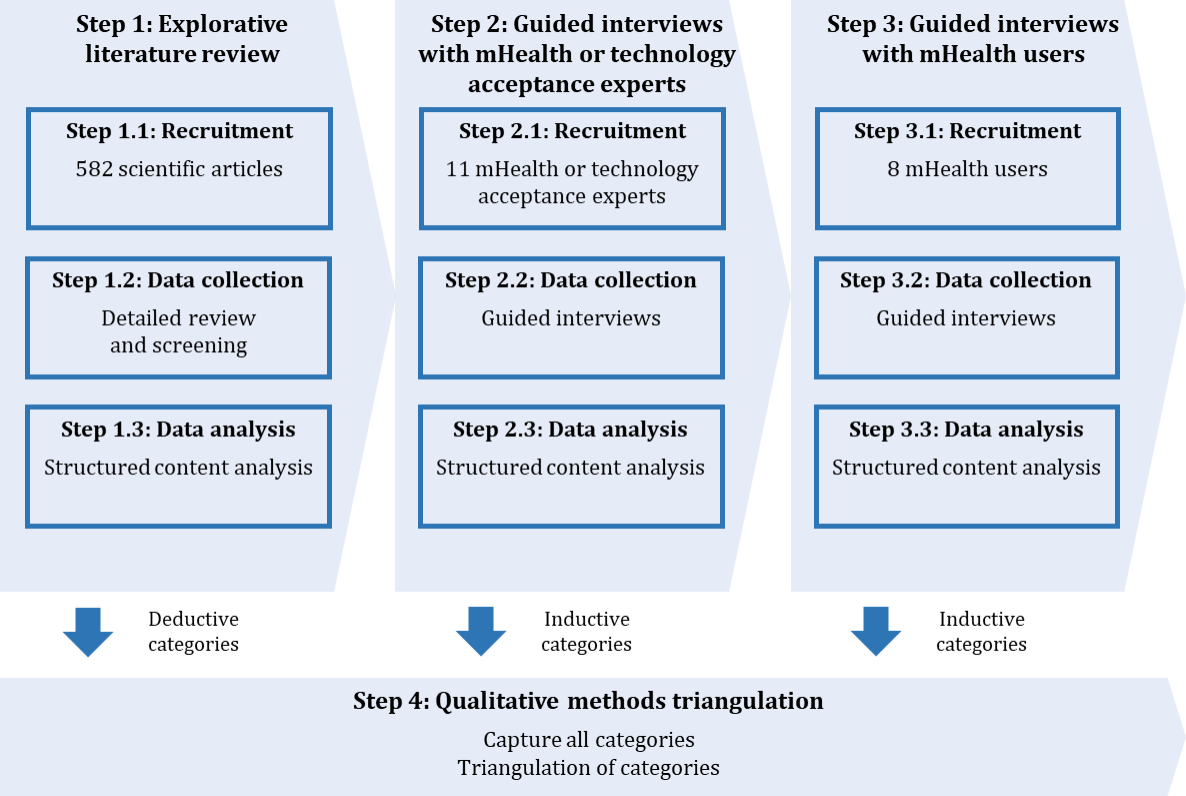
Research design. mHealth: mobile health.

The research design used, as shown in Figure 2, comprises 4 main steps (step 1 to step 4) built on each other. In the first step (step 1), we identified relevant categories from the explorative literature review for the initial category system. In the second step (step 2), we conducted guided interviews with mHealth or technology acceptance experts, followed by the third step (step 3), where we conducted guided interviews with mHealth users. Guided interviews and literature review served to assess the existing exogenous UTAUT2 constructs in a health-related context and identify possible additional categories. In the last step (step 4) of the research process, we used qualitative methods triangulation to capture and compare all identified categories from the previous research steps (step 1 to step 3) and finally confirmed or rejected them.

### Ethics Approval

The study was approved by the research committee for scientific ethical questions of the UMIT Private University for Health Sciences, Medical Informatics and Technology (reference number RCSEQ 2805/20).

### Explorative Literature Review (Step 1)

#### Recruitment (Step 1.1)

Between March and November 2020, we conducted an explorative literature review in the MEDLINE database following systematic criteria. We used the keywords *diabetes* and *diabetes mellitus* for the concept of diabetes. For the concept of mHealth apps, we used the keywords *mobile health apps*, *mobile health applications*, *mobile health units*, and *mobile apps*. For the concept of technology acceptance, we used the keywords *acceptance*, *UTAUT*, and *UTAUT2*. In total, we identified 582 scientific articles using different search queries.

#### Data Collection (Step 1.2)

**Figure 3:**
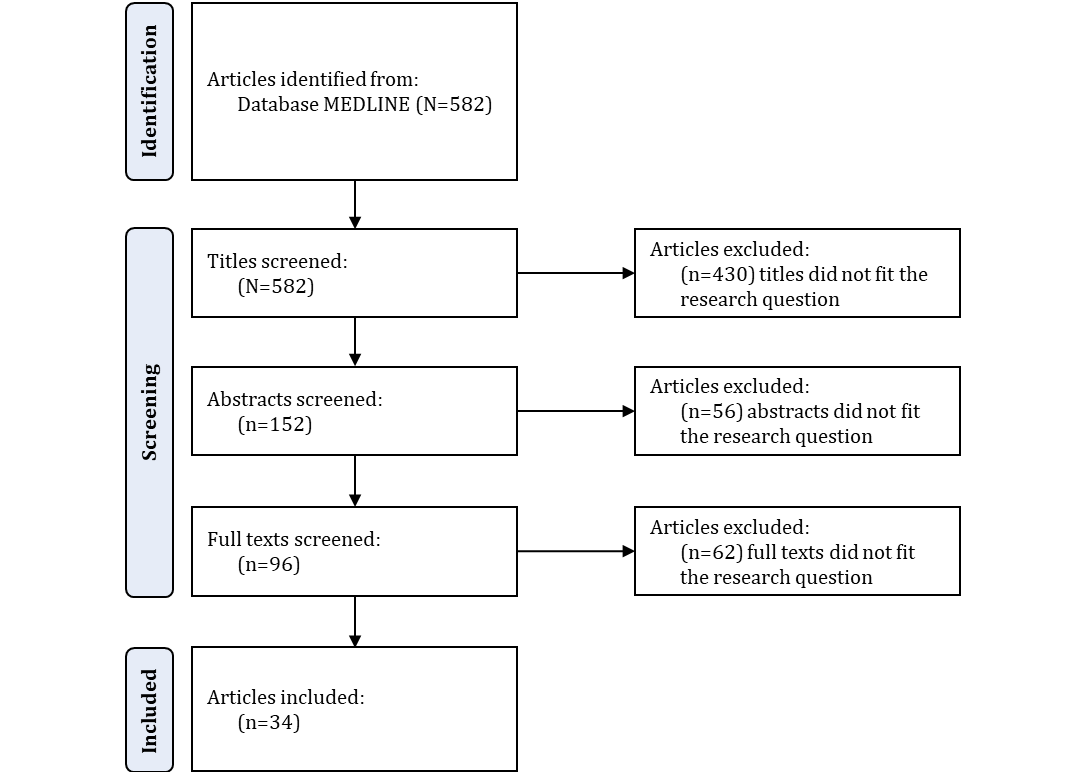
Explorative literature review—the screening process.

The explorative literature review aimed to identify relevant scientific articles from the mHealth and technology acceptance field to develop the initial category system based on the UTAUT2 model and additional categories using diabetes as an example. On the basis of the identified scientific articles, we conducted a screening process, which is described in Figure 3. In the screening process, we first checked the titles and then the abstracts of all scientific articles and compared them with the research question. In these 2 steps, of the 582 scientific articles, we filtered out 486 (83.5%) scientific articles that did not meet the inclusion criteria, and for the remaining 96 (16.5%) scientific articles, we conducted a full-text analysis and compared the content of the methods, results, and conclusions sections with the research question. Approximately 5.8% (34/582) of scientific articles met the inclusion criteria.

#### Data Analysis (Step 1.3)

We conducted data analysis sequentially for each research step (step 1 to step 3). MAXQDA 2020 (release 20.4.0; VERBI GmbH) was used for transcribing and coding the qualitative data. We conducted a structured content analysis using inductive and deductive approaches, following the research question to analyze the qualitative material according to Kuckartz [46]. We developed, used, and continuously updated a codebook containing relevant information (eg, detailed code description and inclusion and exclusion criteria) to ensure the high quality of the coding process [47]. Throughout the complete data analysis process (step 1.3, step 2.3, and step 3.3), we used the method of peer debriefing, in which we critically discussed the collected data and the results derived from that data, as well as the related analysis processes with an experienced research expert [48]. In particular, unclear passages in the qualitative data were reviewed according to the four eyes principle and discussed during meetings with the coauthors to find a shared consensus.

We started the data analysis by coding the scientific articles from the explorative literature review (step 1). In the first deductive step, we defined categories based on exogenous UTAUT2 constructs to develop the initial category system. In the second step, we coded 34 scientific articles based on predefined categories and assigned all the relevant text segments to the corresponding categories. In the third step, we inductively defined and added missing categories to the category system based on the material.

### Guided Interviews With mHealth or Technology Acceptance Experts (Step 2)

#### Recruitment (Step 2.1)

We conducted guided interviews between December 2020 and March 2021 with 11 mHealth or technology acceptance experts (9, 82% men and 2, 18% women) from Germany and Austria. We identified the experts based on their publications and institute websites. Each of the experts held, at minimum, a PhD degree and had worked in the research area of mHealth or technology acceptance for ≥3 years. We used purposive sampling to select the experts from universities in Germany and Austria. After the 11 interviews, theoretical saturation was reached.

#### Data Collection (Step 2.2)

On the basis of the results of the explorative literature review (step 1) and the research question, we developed individual theory-based interview guides with open-ended questions for the interviews with mHealth or technology acceptance experts (step 2) and mHealth users (step 3). We tested and improved the interview guides before the official interviews. The researcher (PS) who conducted the interviews was trained in qualitative research methods and had a positive interest in mHealth apps. There was no personal relationship between the researcher and the interview candidates. The interviews took place only between the researcher and the interview candidate on the web (web conference) or by telephone. Both the researcher and interview candidate were at home or in their own office during the interview; therefore, no one else was present. Before the interview started, there was a short introduction of the researcher, the research topic, and the data privacy guidelines. All interviews were conducted in German, audio recorded by an external audio recording device, and lasted between 20 and 45 minutes. After we finished the interviews, we offered the participants the opportunity to ask questions, which helped improve the interview guides. We took notes on the interview atmosphere and comments from outside the interview. We did not repeat any interviews, and there were no dropouts.

We started the guided interviews with mHealth or technology acceptance experts (step 2). The interviews aimed to assess the existing exogenous UTAUT2 constructs in a health-related context and identify additional relevant categories. Therefore, in the first part of the interviews, we asked questions about the general factors influencing the acceptance and sustained use of mHealth apps from an expert perspective: *which factors significantly affect the acceptance and long-term use of mHealth self-management apps?* In the second part of the interviews, we focused on the UTAUT2 model, specifically on the essential constructs and constructs that should be added based on the experts’ feedback: *which constructs should be supplemented to the UTAUT2 model concerning acceptance investigations of mHealth self-management apps?* (Table 1). For this reason, we adopted an unprompted approach with open-ended questions.

**Table 1:** Main topics of the guided interviews with mHealth^a^ or technology acceptance experts (n=11) and mHealth users (n=8).

mHealth or technology acceptance experts			mHealth users
**General**			
	Factors influencing the acceptance and long-term use of mHealth self-management apps	Factors influencing the (long-term) use of mHealth self-management apps	
	Advantages and disadvantages associated with the use of mHealth self-management apps	Advantages and disadvantages associated with the use of mHealth self-management apps	
	Reasons leading to use or nonuse of mHealth self-management apps	Reasons leading to use or nonuse of mHealth self-management apps	
**Specific**			
	UTAUT2^b^ variables have the most significant influence on the acceptance and use	Expectations, barriers, and emotions related to the use of the mHealth self-management app over time	
	Variables that should be added to the UTAUT2 model to describe the acceptance of mHealth self-management apps	Relevance of the mHealth self-management app in daily life	

^a^mHealth: mobile health.

^b^UTAUT2: Unified Theory of Acceptance and Use of Technology 2.

#### Data Analysis (Step 2.3)

In contrast to the data analysis of the explorative literature review (step 1.3), the analysis of the guided interviews (step 2.3 and step 3.3) was not conducted at the end of the entire data collection phase but continuously after each interview. This iteration process helped us identify the point of theoretical saturation; that is, the point at which we were no longer able to identify new categories [48].

In the first step, we continued the data analysis by transcribing the guided interviews with mHealth or technology acceptance experts verbatim. We did not return the interview transcripts to the participants. In the second step, we coded each interview based on the differentiated category system containing the deductive and inductive categories, which resulted from the explorative literature review (step 1.3). In the third step, we inductively defined and added missing categories to the category system based on the material until saturation was reached.

### Guided Interviews With mHealth Users (Step 3)

#### Recruitment (Step 3.1)

Between March and May 2021, we conducted guided interviews with 8 mHealth users (5, 63% men and 3, 38% women) from Germany and Austria. The age distribution of the participants ranged from 20 to 75 years. We included patients with type 1 and type 2 diabetes and parents caring for children with type 1 diabetes, as the requirements and needs for mHealth apps are comparable, and the apps do not specifically address only one user group. We only included participants using an mHealth app (diabetes app and CGM system) for at least 3 months.

We identified mHealth users through gatekeepers in organizations such as diabetes associations and diabetes self-help groups, who asked suitable persons to participate in the study. In addition, we published a call for participation in the study on social media. We used purposive sampling to recruit patients of different ages, genders, and socioeconomic backgrounds to ensure a wide diversity (Table 2). After 8 interviews, theoretical saturation was reached.

**Table 2:** Sociodemographic data of recruited mHealth^a^ users.

User	Age (years)	Gender	Education	Residence	Type of diabetes	Duration of mHealth app use
1	75	Female	PhD	Austria	Type 2	4 months
2	33	Female	Vocational qualification	Germany	Type 1	4 years
3	52	Male	PhD	Austria	Type 2	6 months
4	20	Female	Vocational qualification	Germany	Type 1	2 years
5	40	Male	PhD	Austria	Father of type 1 diabetes child	4 years
6	22	Male	Student	Germany	Type 1	3 years
7	23	Male	Student	Austria	Type 1	6 years
8	60	Male	Master’s	Austria	Type 2	4 months

^a^mHealth: mobile health.

#### Data Collection (Step 3.2)

After we completed all the interviews with mHealth or technology acceptance experts (step 2), we continued the interviews with mHealth users (step 3). The interviews also aimed to confirm the existing exogenous UTAUT2 constructs and identify additional relevant categories based on mHealth users’ perspectives. The first part of the interviews focused on the used mHealth app and the reasons for choosing and using it: *which features or functions are essential to you so that you use diabetes mHealth self-management apps in the long term?* In the second part of the interviews, we focused on the users’ experience with the mHealth app: *when you first started to use diabetes mHealth self-management apps, what expectations did you have?* (Table 1). For this purpose, we used the user experience (UX) curve method [49], which visualized the UX throughout use. We drew the UX curve by sharing the screen with the mHealth users who joined the interviews on the web. This was not possible if the interview was conducted via telephone. In those cases, we only asked questions on UX without visualization using the UX curve method.

#### Data Analysis (Step 3.3)

To analyze the data from the mHealth user interviews (step 3.3), we performed the same analysis steps as for the analysis of the mHealth or technology acceptance expert interviews (step 2.3). However, for coding the mHealth user interviews, we used the already differentiated category system that included inductive categories from the interviews with mHealth or technology acceptance experts (step 2.3). Approximately 2 weeks after the coding of all material from step 1 to step 3 was completed, we reviewed the final category system and the coded segments to ensure the reliability (intrarater reliability) of the analyzed data [50]. Identical or similar categories were combined.

### Qualitative Methods Triangulation (Step 4)

We used qualitative methods triangulation [51,52] to combine the different perspectives from the explorative literature review (step 1) and the guided interviews (step 2 and step 3) to investigate the research question, thereby increasing confidence in the results and their validity [48,52]. For this purpose, we captured and compared all identified categories from the 3 research steps (step 1 to step 3) to determine the relevant categories to answer the research question. We considered categories that we identified in at least two of the three research steps (step 1 to step 3) to be particularly important for extending the UTAUT2 model.

## Results

### Overview

**Figure 4:**
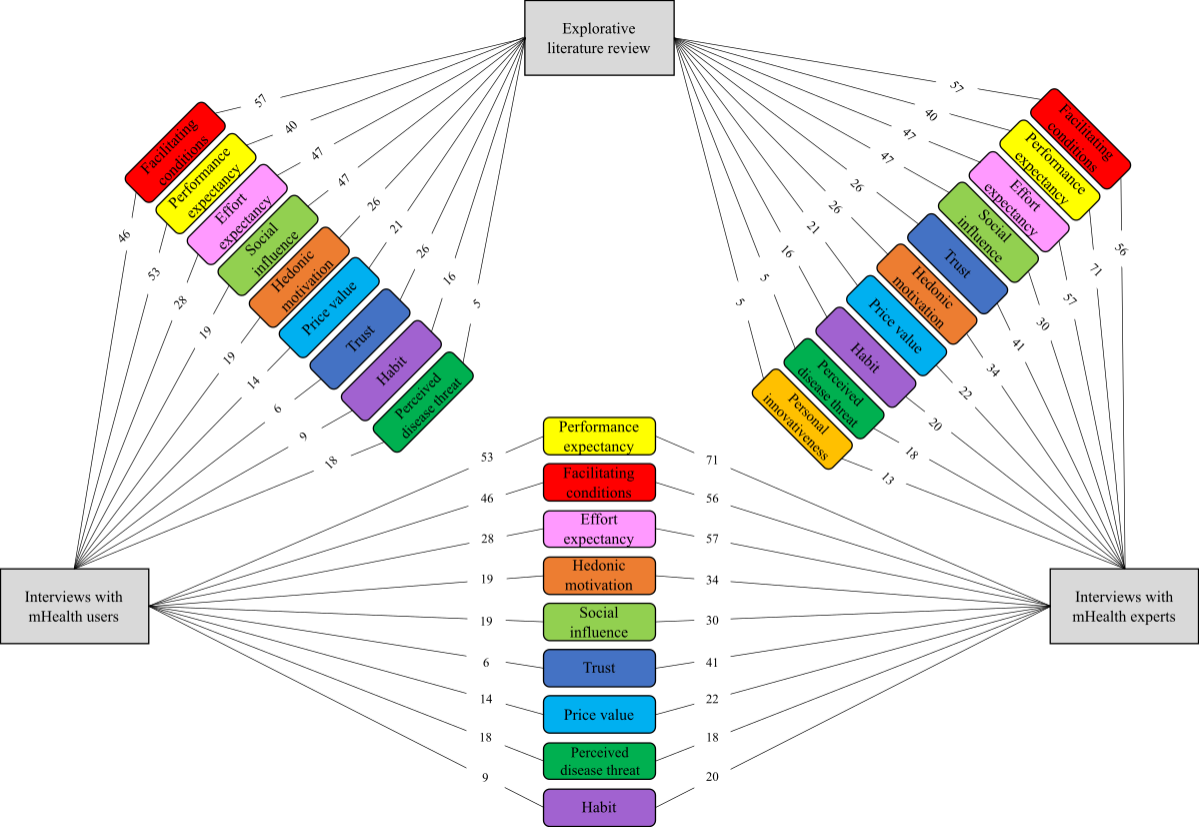
Summary of combined categories (colored boxes) identified from explorative literature review and guided interviews (gray boxes). The figures between gray and colored boxes indicate the number of coded segments assigned to each category. Categories are arranged in decreasing order according to the sum of coded segments from both sources. mHealth: mobile health.

We conducted a qualitative methods triangulation study comprising an explorative literature review (step 1) and guided interviews with 11 mHealth or technology acceptance experts (step 2) and 8 mHealth users (step 3). Using diabetes as an example, we investigated whether the UTAUT2 model is suitable for predicting mHealth acceptance. Thus, we analyzed the material from the explorative literature review (step 1) and the guided interviews (step 2 and step 3) using structured content analysis and then combined the results using qualitative methods triangulation (step 4), as shown in Figure 4.

In our qualitative methods triangulation study, we were able to confirm the relevance of all exogenous UTAUT2 constructs in predicting mHealth acceptance using diabetes as an example. In addition, we were able to identify another three categories that are not part of the UTAUT2 model: trust, perceived disease threat, and personal innovativeness.

Interview quotes translated verbatim are shown in the following sections to support our results.

### Confirmation of Exogenous UTAUT2 Constructs

#### Overview

According to most (9/11, 82%) of the mHealth or technology acceptance experts interviewed, the UTAUT2 model is suitable for acceptance studies, especially in areas where motivation and continuous and voluntary use are essential. For example, this applies to mHealth self-management in diabetes:

...in my opinion, it can be used well for all things that are based on voluntariness in the broadest sense...that means especially with health apps that are not compulsory...even if the doctor prescribes me the app free of charge that does not mean that I will use it for the next weeks and months...

Expert 3, male

Therefore, as a first essential part of our study, we wanted to confirm that the exogenous UTAUT2 constructs are suitable for predicting mHealth acceptance using diabetes as an example. We summarized the main results of the structured content analysis focusing on the exogenous UTAUT2 constructs to present their relevance in the following sections.

#### Facilitating Conditions

We were able to confirm the importance of facilitating conditions in diabetes mHealth self-management. In the explorative literature review, we identified several scientific articles that pointed out the relevance of facilitating conditions in mHealth self-management [5,18,21,53-58]. In particular, the authors highlighted technical support, support from the mHealth app itself, and health care professionals as the essential aspects of facilitating conditions for mHealth apps. We were also able to identify those aspects in the guided interviews, as shown in the extracts in the following section.

According to all (8/8, 100%) of the mHealth users surveyed, good technical support, especially if there are any problems, and support from their medical physician are essential for accepting mHealth apps:

...it is crucial to me in any case, especially with technical problems when the sensor, the mechanism is broken, that you are told how to place it or that you can easily contact the support...In any case, it is vital to me that the doctors can get a good picture and simply that the disease is kept under control.

User 7, male, type 1 diabetes

Some (4/11, 36%) of the mHealth or technology acceptance experts confirmed that support from medical physicians is an essential factor for the long-term use of mHealth self-management apps:

...many people also want to have some kind of connection with their doctor. The app is used or recommended by the doctor, or the doctor can be contacted if there are any questions. So this is not just pure self-help, but there is some connection with the healthcare system or with health service...

Expert 9, female

Of course, the support from the mHealth app itself is also essential for its use. Some (3/8, 38%) of the mHealth users consider a decent help function (eg, frequently asked questions and video tutorials) that facilitates the use of the mHealth self-management app to be an essential feature:

...with pictures and text, there are even videos...every time you put a new sensor...you get an explanation how to do it...

User 4, female, type 1 diabetes

#### Performance Expectancy

We were also able to confirm the importance of performance expectancy in diabetes mHealth self-management. On the basis of the explorative literature review, we identified several scientific articles that showed the importance of performance expectancy in the context of mHealth self-management [18,21,22,55,59-61]. In particular, the authors highlighted the benefits gained for disease management as a relevant aspect of mHealth apps. We were also able to identify this aspect of performance expectancy in the guided interviews, as shown in the following sections.

Most (7/8, 88%) of the mHealth (CGM system) users started using the technology because of clear expectations that it would improve their lives. The mHealth app makes daily management of the disease easier and gives users back a piece of everyday life as it provides an accessible overview of the relevant blood glucose values, which has a positive effect on acceptance:

...we switched to it because it is simply a completely different dimension in diabetes management. You can not compare that with regular blood measurements...with this technology, diabetes is just much easier to handle. You can go about your daily life...

User 5, male, father of type 1 diabetes child

Compared with traditional blood glucose monitoring, half (4/8, 50%) of the surveyed mHealth (CGM system) users considered setting alarms and reminders in urgent situations to be one of the most crucial functions of the mHealth app, thus increasing its acceptance:

...definitely the alarms. I have an Apple Watch that is also compatible with the system. It gives me messages...when I am hypoglycemic when there are any disorders. What I also find to be a significant advantage is sharing the app with other people. So my partner also has it on his cell phone and sees or gets messages when I am hypoglycemic and can no longer react...

User 4, female, type 1 diabetes

This also reflects the statements of all (11/11, 100%) of the mHealth or technology acceptance experts. The essential factors for long-term use of mHealth self-management apps are the perceived benefits and advantages that must be visible to the patients, especially the freedom gained and the flexibility in self-management of the disease:

...so people have to see that they have a benefit somehow. So over a longer period, they also use it consistently in everyday life...that is the case with chronic diseases, where it is a long-term problem, and you have to make people aware of the benefits of this app for the long term. It must be useful...

Expert 9, female

#### Effort Expectancy

We were also able to confirm the importance of effort expectancy in diabetes mHealth self-management. In the explorative literature review, we were able to identify several scientific articles that showed the importance of effort expectancy in the context of mHealth self-management [5,18,21,40,53,55,57,59,61,62]. In particular, the authors highlighted convenience, simplicity, and usability as relevant aspects associated with the acceptance and use of mHealth apps. As shown in the extracts given in the following section, we were also able to identify those aspects of effort expectancy in the guided interviews.

Usability and simplicity of use without physical impairment were deemed to be essential criteria for long-term use of diabetes mHealth self-management apps by all (8/8, 100%) of the mHealth users. A relevant aspect that half (4/8, 50%) of the surveyed mHealth (CGM system) users highlighted is that drawing blood is not necessary for the glucose measurements, which is a great relief in everyday life and improves acceptance:

...it is just a lot easier than when you have to go to the break room at work all the time and prick your finger...take your cell phone, hold it up to the sensor, and it shows you the sugar right away...it is just a significant relief, and you have everything in there...you do not have to keep a diary anymore. You have everything in the app. Everything is there...

User 2, female, type 1 diabetes

In all (11/11, 100%) of the mHealth or technology acceptance experts’ point of view, the decisive factors for the long-term use of mHealth apps are their user-friendliness and the fact that they require less effort, are easy to use, and produce better outcomes than conventional solutions:

...especially the usability plays a decisive role...

Expert 6, male

#### Social Influence

We were also able to confirm the importance of social influence in diabetes mHealth self-management. On the basis of the explorative literature review, we identified several scientific articles that showed the importance of social influence in the context of mHealth self-management [5,18,20-22,36,40, 53,57,59,63]. In particular, the authors highlighted the importance of recommendations from physicians, medical professionals, family members, and friends for the use of mHealth apps. We were also able to identify those aspects in the guided interviews, as shown in the extracts in the following sections.

On the basis of feedback from all (8/8, 100%) of the mHealth users, the primary influence to use an mHealth app for disease management is driven by health care providers such as diabetologists, diabetes outpatient clinics, and physicians. If the personal environment is generally very positive about the mHealth app, acceptance is encouraged:

...I was only really made aware of this by my diabetologist. So through her, I got to know that, before I did not know that either...

User 2, female, type 1 diabetes

In addition, many (8/11, 73%) of the mHealth or technology acceptance experts see significant influence from health care providers for the first and long-term use of mHealth apps. Most (9/11, 82%) of the mHealth or technology acceptance experts also see some influence from the media and closer personal environment, which positively influences acceptance:

...so the recommendation by a doctor, by friends, relatives or other persons involved is fundamental...

Expert 9, female

#### Hedonic Motivation

We were also able to confirm the importance of hedonic motivation in diabetes mHealth self-management. On the basis of the explorative literature review, we were able to identify several scientific articles that showed the importance of hedonic motivation in the context of mHealth self-management [18,22,56,59,62]. In particular, the authors emphasized the importance of emotional support for adherence, motivation through goal setting, and playful elements (ie, gamification) for the sustained use of mHealth apps. We were also able to identify those aspects in the guided interviews, as shown in the extracts given in the following section.

All (8/8, 100%) of the mHealth users reported positive emotions, such as the joy of having an app that helps them manage their disease. Some (5/8, 63%) of the mHealth users associate the use of the app with fun, which leads them to check blood glucose much more frequently, for example, which contributed to increasing the acceptance of the mHealth app:

...first of all, joy, because it is a significant relief...

User 2, female, type 1 diabetes

...you have something new,...you want to use it all the time although it is a medical application...I measured blood sugar fifty times a day...just to see how cool it is...

User 6, male, type 1 diabetes

In addition, most (9/11, 82%) of the mHealth or technology acceptance experts consider fun, such as through gamification aspects and positive feedback during use, to be vital motivating factors for ensuring that mHealth apps are used for the long term:

...hedonic motivation plays a role—of course, it is a decisive factor in whether you use it or not...I also enjoy it...I find gamification exciting, i.e., increasing motivation through such playful elements...

Expert 5, male

#### Price Value

We were also able to confirm the importance of price value in diabetes mHealth self-management; however, we also identified 2 levels. On the basis of the explorative literature review, we discovered that depending on the user group (eg, older patients) or the mHealth app (eg, sensors with higher costs), price value played a significant role [18,61,63]. In contrast, the price was not relevant for less expensive mHealth apps such as smartphone apps [22,53,59]. We were also able to identify those aspects in the guided interviews, as shown in the extracts given in the following section.

Approximately all (7/8, 88%) of the mHealth users assigned a rather subordinate role to the price, especially for mHealth smartphone apps. Their focus was on the gain in convenience and quality of life. Health insurance companies usually cover the costs of the considerably more expensive CGM systems. However, the mHealth users agreed that even an appropriate copayment would not affect the use and acceptance of the system:

...so I do not have to pay anything for the smartphone app. I just need to pay for the sensors. So that is thirty euros a quarter, which is nothing. Even if the app had to be paid for, it depends on how much, of course...I would definitely pay...because it is a significant relief and would be worth it to me...

User 2, female, type 1 diabetes

If the price of an mHealth self-management app is within a reasonable range, most (9/11, 82%) of the mHealth or technology acceptance experts consider it to have no significant role. However, if the price is too high, it will affect acceptance, and people will not start or continue using the mHealth app:

...the price is often unimportant because the things are either free or paid for by the health insurance—so, in the very rarest cases, I have to spend a large amount of money for a specific application...

Expert 3, male

#### Habit

We were also able to confirm the importance of habit in diabetes mHealth self-management. On the basis of our explorative literature review, we identified several scientific articles that showed relevant aspects of habit in the context of mHealth self-management [53,59,62,64]. The authors emphasized the strong influence of habit on the expected outcome and the importance of continuous use because of regular patterns and routines. We were also able to identify those aspects in the guided interviews, as shown in the extracts given in the following section.

Approximately all (7/8, 88%) of the mHealth users stated that the mHealth app has taken an important place in their everyday life and has become a habit, improving their disease management:

...important place in my life. Compared to before, now with the app I test, I think, almost fifteen times more than before. It is already routine...I test much more than before with a standard test device...

User 2, female, type 1 diabetes

In addition, approximately half (5/11, 45%) of the mHealth or technology acceptance experts confirmed that integrating the mHealth app into daily routines is essential for sustained use:

...the habit is, of course, what drives you in the end, to do the same thing over and over again...

Expert 4, male

### Additional Constructs

However, the analysis showed that additional constructs, as shown in Textbox 2, may also need to be considered to predict the user acceptance of mHealth self-management apps in diabetes.

**Newly proposed and confirmed constructs for the acceptance of mobile health self-management in diabetes.**

**Trust**

- Degree of trust in the data collected by the mobile health app concerning data security, privacy, quality, and processing

**Perceived disease threat**

- Degree of patients’ awareness of risks and limitations to health and well-being related to diabetes

#### Newly Proposed and Confirmed Construct: Trust

We identified the construct *trust* in several places in this study, showing its relevance to the field of mHealth self-management in diabetes. Trust can be defined as belief or confidence in other people or things [65]. We used the term *trust* to combine aspects such as data security, privacy, anonymity, and information quality. This approach is in line with a recent study on public trust in the health care system, in which the authors investigated various aspects that influence trust to understand the construct better [66]. On the basis of the explorative literature review, we identified several scientific articles that highlighted the importance and relevant aspects of trust in the context of mHealth self-management [18,21,22,40,59,65,67]. The authors emphasized the positive influence of trust as a crucial aspect in predicting acceptance and intention to use IT. We could also identify these aspects in the guided interviews, as shown in the extracts given in the following section.

Data protection and privacy were considered essential features of mHealth self-management apps by half (4/8, 50%) of the surveyed mHealth users. Problems with insufficient data protection and privacy can lead to low acceptance and termination of use:

...possibly lead to the fact that I stop...if I have the feeling my privacy is not maintained...

User 1, female, type 2 diabetes

In addition, from the perspective of most (10/11, 91%) of the mHealth or technology acceptance experts, data protection, data security, and privacy are central prerequisites for the acceptance and long-term use of mHealth apps. Specifically, the handling of data by third parties, such as service providers, has a significant influence on the use decision of mHealth apps:

...you also have to trust the app provider or manufacturer...if there is even the slightest risk that personal data is sold, and not anonymized at best...this does not increase trust, and the application probably will not be used...

Expert 5, male

Thus, according to more than half (7/11, 64%) of the surveyed mHealth or technology acceptance experts, it is not only about technical parameters of the mHealth app, such as data security. They increasingly see subjective factors such as trust in the service and service provider as relevant for mHealth acceptance:

...data protection is only one aspect...it is really about trust...[Expert 5, male]

As the construct *trust* is not part of the UTAUT2 model but essential for accepting and using mHealth self-management apps, some (5/11, 45%) of the mHealth or technology acceptance experts recommended adding it:

...something like the trust that the data is not being misused...it is such a central aspect...because trust is, at least in Germany and I also think in Austria...a central component of consumer health IT applications.

Expert 5, male

Therefore, we were able to confirm the importance of trust for mHealth self-management in diabetes.

#### Newly Proposed and Confirmed Construct: Perceived Disease Threat

In this study, we identified the construct *perceived disease threat* in several places. The Health Belief Model first defined the construct of *perceived disease threat* [68,69]. The Health Belief Model refers to avoiding and preventing illness through specific health actions [70]. In this study, we used the construct of *perceived disease threat* to assess patients’ awareness of risks and limitations to health and well-being associated with diabetes [70]. On the basis of our explorative literature review, we identified several scientific articles that highlight the importance and relevant aspects of perceived disease threat in the context of mHealth self-management [19,21,34,40,71]. In particular, the authors highlighted that patient awareness of the risks associated with chronic diseases could help to improve the acceptance of mHealth self-management apps. We were also able to identify those aspects in the guided interviews, as shown in the extracts given in the following section.

Many (5/8, 63%) of the mHealth users mentioned that they started to use a CGM system because of the negative impact on their blood glucose levels when they partially stopped using the conventional measurement because of the perceived inconvenience of pricking their finger. In addition, the CGM system protects against dangerous situations such as nighttime hypoglycemia, which they highlighted to be essential for acceptance:

...the pricking was highly burdensome to me, so I partly stopped doing it, which was not really beneficial for developing blood glucose levels...

User 3, male, type 2 diabetes

...you never know what happens at night when you do not wake up when you have hypoglycemia, and if I didn’t have the system, quite different things could happen...

User 4, female, type 1 diabetes

According to more than half (6/11, 55%) of the surveyed mHealth or technology acceptance experts, people who experience a disease and perceive it as a risk are more open to alternatives such as mHealth apps that promise positive benefits, which increases their acceptance:

...the patients’ current state of health and suffering are essential...someone who has to ensure very extensive self-management is much more open-minded than someone who only has to collect or document data once a day or once a week...

Expert 10, female

Therefore, according to some (3/11, 27%) of the mHealth or technology acceptance experts, the UTAUT2 model should be extended with variables related to the disease state and the perceived disease threat:

...I would include disease-related variables...for example, chronic diseases...something like a perceived threat.

Expert 9, female

Therefore, we were able to confirm the importance of the perceived disease threat for mHealth self-management in diabetes.

#### Newly Proposed but Not Confirmed Construct: Personal Innovativeness

We were able to identify the construct of *personal innovativeness* only in the explorative literature review and the interviews with mHealth or technology acceptance experts but not with mHealth users. On the basis of the explorative literature review, we identified only 2 articles that highlighted the importance of personal innovativeness in the context of mHealth self-management [59,60]. The authors described personal innovativeness as the ability of a person to be open to new ideas and make innovative decisions [60]. As shown in the extract given in the following section, we were also able to identify the described aspect in the interviews with mHealth or technology acceptance experts.

Most (8/11, 73%) of the mHealth or technology acceptance experts see technology-savvy people and people who want to control their data as being particularly open to accepting and using mHealth apps:

...especially technically-savvy patients, as well as patients who do not want to travel to the hospital three times a week to record a certain value...

Expert 6, male

However, the construct is already part of the moderating effects of hedonic motivation on behavioral intention in the UTAUT2 model because of the associated differences in users’ willingness to innovate [26]. Therefore, we were not able to confirm the importance of personal innovativeness as an additional construct for mHealth self-management in diabetes.

## Discussion

### Principal Findings

In this qualitative methods triangulation study, we used different perspectives to investigate whether the UTAUT2 model is suitable for predicting mHealth acceptance using diabetes as an example. Our results showed that we were able to confirm all exogenous UTAUT2 constructs. However, we verified that 2 essential constructs are missing in the UTAUT2 model to predict mHealth acceptance. We determined the constructs of *trust* and *perceived disease threat* to be relevant in this context. In contrast, the construct *personal innovativeness*, which we also identified, seemed less relevant for mHealth users, as we did not find indicators in the interviews. Furthermore, the construct *personal innovativeness* is already considered in the UTAUT2 model; therefore, it is unnecessary to add it as a separate construct.

### Strengths and Limitations

We used a qualitative research method with its open approach to investigate the subject area.

The triangulation of explorative literature review (step 1) and guided interviews with mHealth or technology acceptance experts (step 2) and mHealth users (step 3) allowed us to identify relevant aspects influencing mHealth acceptance from different perspectives.

Using the method of structured content analysis combined with qualitative methods triangulation allowed us to confirm all relevant categories. In addition, we were able to identify less relevant categories; therefore, those categories are not required to be added to the UTAUT2 model. As expected, the systematic combination of the different methods proved successful, as we were able to confirm all exogenous UTAUT2 constructs and identify new categories quite clearly. The results have confirmed each other and can, therefore, be considered reliable.

We chose diabetes as an example as it is one of the most common chronic diseases for which mHealth apps are an essential element of self-management. Owing to the broad spectrum of patients with diabetes and available mHealth apps, the qualitative results also seem to be generalizable to mHealth apps for other chronic diseases.

We systematically selected different interview participants and triangulated different sources of information. We also followed the principle of theoretical saturation and are, therefore, confident that we have captured all relevant aspects. However, as the selection of mHealth users focused on active users, there might have been some selection bias.

Although the interviews were only conducted with people from Austria and Germany using diabetes as an example, we consider that the qualitative methods triangulation study results also apply to countries with comparable health care systems, technical infrastructure, socioeconomic and cultural backgrounds, and other chronic diseases where mHealth self-management apps are used because of the multicenter study design.

A risk when conducting interviews is that people’s responses may be influenced by social desirability. We tried to reduce this risk by creating a trustful and open interview atmosphere in which only the interviewer and the interview candidate were present.

Although we have adhered to the quality criteria of qualitative research concerning objectivity, reliability, and validity by applying neutrality in data analysis, rule guidance in the research process, peer debriefing, and method triangulation, explorative studies are associated with certain limitations such as generalizability. Therefore, we plan to verify the results within the framework of a quantitative follow-up study.

### Comparison With Prior Work

To date, there have not been many studies that have used the UTAUT2 model to predict mHealth acceptance [41,43,44,60,62,64]. In addition, only a few of these studies have explicitly highlighted the suitability of the UTAUT2 model in this context [43,60,64]. With our qualitative methods triangulation study, we were also able to confirm the suitability of the UTAUT2 model for predicting mHealth acceptance.

In our results, we showed that the four exogenous UTAUT constructs of *facilitating conditions*, *performance expectancy*, *effort expectancy*, and *social influence* are relevant to the acceptance of mHealth in diabetes, which is consistent with previous mHealth studies [19,36,37,41].

We were also able to verify the relevance of the three additional exogenous UTAUT2 constructs: *hedonic motivation*, *price value*, and *habit*. In particular, *hedonic motivation* and *habit* were highlighted to be essential for the acceptance and long-term use of mHealth self-management apps in diabetes. In their study, the authors pointed out the importance of both constructs for mHealth acceptance [64].

Our results showed that the price of an mHealth self-management app is considered less relevant by mHealth or technology acceptance experts and mHealth users, who focus more on the benefits of the app. This observation is consistent with the findings from previous studies, where the authors showed that price value does not influence mHealth acceptance [43,59].

In addition to the exogenous UTAUT2 constructs, we identified three relevant constructs: *trust*, *perceived disease threat*, and *personal innovativeness*. The relevance of *trust* and *perceived disease threat* were highlighted in our results as essential aspects for mHealth acceptance in diabetes. This observation aligns with previous studies where the authors described the relevance of *trust* in adopting different eHealth services by extending the TAM and UTAUT model [65,67].

The relevance of the construct *perceived disease threat* was also confirmed by several studies where the authors used the TAM and UTAUT model to investigate the acceptance and adoption of mHealth apps in patients with chronic diseases such as hypertension and diabetes [19,21,34,71].

However, our results showed that the construct of *personal innovativeness* turned out to be less relevant. This observation is consistent with the original UTAUT2 study in which the authors described the construct *personal innovativeness* as an implicit moderating effect of the construct *hedonic motivation* on *behavioral intention* [26].

### Conclusions

In summary, our study showed that the UTAUT2 model is suitable for predicting mHealth acceptance, as shown in the field of mHealth for diabetes. However, we also showed that the additional constructs of *trust* and *perceived disease threat* are required to comprehensively examine mHealth acceptance in this context.

We see great potential for an extended UTAUT2 model that focuses on additional mHealth predictors. Further research is needed to determine whether the newly identified constructs also apply to other mHealth apps and clinical settings.

## Abbreviations

CGM: continuous glucose monitoring
COREQ: Consolidated Criteria for Reporting Qualitative Research
IT: information technology
mHealth: mobile health
TAM: Technology Acceptance Model
UTAUT: Unified Theory of Acceptance and Use of Technology
UTAUT2: Unified Theory of Acceptance and Use of Technology 2
UX: user experience
